# Omics-Based Biomarkers: Application of Metabolomics in Neuropsychiatric Disorders

**DOI:** 10.1093/ijnp/pyv096

**Published:** 2015-10-09

**Authors:** Sumit Sethi, Elisa Brietzke

**Affiliations:** Interdisciplinary Laboratory for Clinical Neuroscience (LiNC), Department of Psychiatry, Universidade Federal de São Paulo - UNIFESP, São Paulo, Brazil.

**Keywords:** biomarkers, bipolar disorder, drug addiction, metabolomics, schizophrenia

## Abstract

One of the major concerns of modern society is to identify putative biomarkers that serve as a valuable early diagnostic tool to identify a subset of patients with increased risk to develop neuropsychiatric disorders. Biomarker identification in neuropsychiatric disorders is proposed to offer a number of important benefits to patient well-being, including prediction of forthcoming disease, diagnostic precision, and a level of disease description that would guide treatment choice. Nowadays, the metabolomics approach has unlocked new possibilities in diagnostics of devastating disorders like neuropsychiatric disorders. Metabolomics-based technologies have the potential to map early biochemical changes in disease and hence provide an opportunity to develop predictive biomarkers that can be used as indicators of pathological abnormalities prior to development of clinical symptoms of neuropsychiatric disorders. This review highlights different -omics strategies for biomarker discovery in neuropsychiatric disorders. We also highlight initial outcomes from metabolomics studies in psychiatric disorders such as schizophrenia, bipolar disorder, and addictive disorders. This review will also present issues and challenges regarding the implementation of the metabolomics approach as a routine diagnostic tool in the clinical laboratory in context with neuropsychiatric disorders.

## Introduction

Millions of people undergo mental disorders such as major depressive disorder (MDD), bipolar disorder (BD), schizophrenia (SCZ), and addiction. According to the World Health Organization, the worldwide problem of neuropsychiatric disorders is 13% higher than others such as cardiovascular diseases and cancer (WHO, 2008). Though significant improvement has been made in the treatment of neuropsychiatric disorders, numerous patients do not respond to current therapies, had an inadequate response, or are incapable to tolerate them.

Unfortunately, our understanding of pathophysiology of these disorders remains limited. One reason for this is the fact that most of mental disorders are not unitary conditions but may be a complex of psychopathological dimensions that are yet to be identified. In addition, present knowledge is also incomplete in predicting who will and who will not respond to a certain treatment. Such doubtfulness is worrying for patients and families who are continually involved in trial-and error selections in search of “the right fit” and for clinicians thus resorting to extensive substituting of medications (Weiden and Buckley, 2007) and polypharmacy (Tranulis et al., 2008). So, there is a further requirement to scale up awareness in the study of psychiatric disorders in an effort to recognize at a system level the entirety of alterations that can contribute to the pathogenesis of these environments. Disease-specific molecular fingerprinting can be well-defined by integrating the use of high-throughput methodologies at the core of genomics, proteomics, metabolomics, and other -omics approaches and could aid to map dysregulated systems involved in disease pathogenesis. Furthermore, global mapping of uncharacteristic pathways in psychiatric disorders can lead to the identification of biomarkers of disease and response (Quinones and Kaddurah-Daouk, 2009).

This review summarizes general aspects of biomarker research and how metabolic abnormalities in psychiatric disorders can contribute to the identification of distinctive biomarkers. We also discuss existing challenges and the potential of metabolic approaches in the process of biomarker discovery.

## Biomarker Discovery Research

The use of the term “biomarker” dates back to as early as 1980 (Jeffrey, 2005). In 1998, the National Institutes of Health Biomarkers Definitions Working Group described a biomarker as “a characteristic that is objectively measured and evaluated as an indicator of normal biological courses, pathogenic progressions, or pharmacologic responses to a therapeutic intervention” (Aronson, 2005; Strimbu and Tavel, 2010). In metabolomics, biomarkers can be measured in any biological sample, for example, blood, urine, or saliva (Bogdanov et al., 2008; Holmes et al., 2008b; Kaddurah-Daouk et al., 2009) and can be indicators of disease traits (or risk markers), disease states, or disease rates (progression).

**Figure 1.**
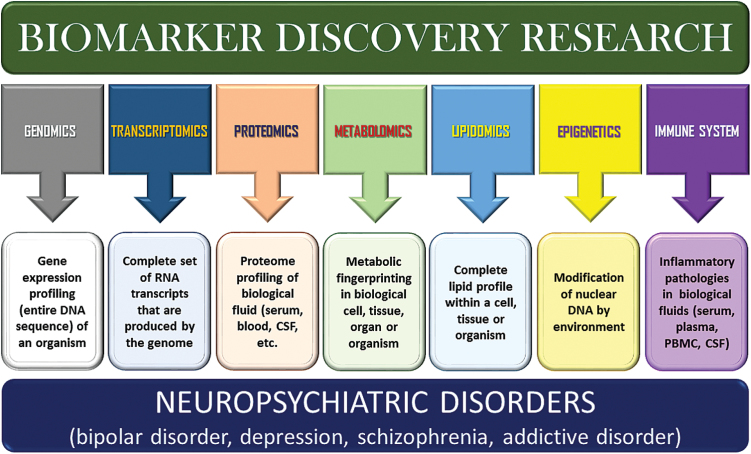
Schematic representation of various biological approaches for biomarker discovery in neuropsychiatric disorders.

Biomarkers could be considered to extend all the way to include our fixed genomic characters. At the level of the subcellular and tissue, the search has queried the transcriptomics, proteomics, metabolomics, lipidomics, immunological, and biological epigenetics (Figure 1). The recent attention in biomarker discovery is encouraged by new molecular biologic techniques with the ability to find relevant markers speedily without detailed perception into the mechanisms of a disease.

### Biomarkers in Genomics

Mental disorders include a wide spectrum of diseases in the central nervous system (CNS), with symptoms varying from cognitive dysfunction to alterations in emotions, thoughts, or performance (Krystal and State, 2014). Due to the complex nature of brain disorders, it is unfruitful to find out the mechanisms using conventional methodologies, where only small pathways around specific target genes are examined. Recently, genomic technologies have been progressively applied to the investigation of neuropsychiatric disorders (McCarroll et al., 2014). Remarkably, genome-wide association studies (GWAS) have significantly increased the knowledge of the genetic basis of psychiatric disorders (Ozomaro et al., 2013; Luykx et al., 2015). GWAS are based on the fact that several single-nucleotide polymorphisms (SNPs) in a defined haplotype provide the same information for association with a causal variant, thus decreasing SNPs to be tested for association. As most of the common SNPs are situated in genomic regions with no clear function (e.g., intronic areas or intergenic regions), identification of the fundamental “causal” variant or the identification of the related function needs further efforts. Many of the SNPs have been associated with predicting treatment response (both in terms of therapeutic efficacy and side-effect profile) to a pharmacologic intervention. One of the challenges for a successful GWAS in the future will be to apply the findings in a way that accelerates drug and diagnostics development as well as better integration of genetic studies into the drug-development process and a focus on the role of genetic variation in maintaining health as a blueprint for designing new drugs and diagnostics (Iadonato and Katze, 2009).

Lymphocyte gene expression profiling has emerged as a predominantly interesting area of research in the examination for peripheral biomarkers (Tsuang et al., 2005). Many studies have focused on human blood gene expression profiling, comparison between illness and healthy control groups, and cross-matching with human postmortem brain gene expression data (Le-Niculescu et al., 2007). Iga et al. (2007) studied peripheral gene expression before and after treatment of major depressed patients and reported high levels of histone deacetylase 5 and cyclic-AMP response element binding protein 1 prior to treatment, with a significant decrease following 8 weeks of antidepressant treatment. de Jong et al. (2012) did a large peripheral gene expression study with medicated SCZ subjects (n=92), unmedicated SCZ (n=29), and 118 healthy controls. They focused on determining coexpression networks related with SCZ, nevertheless of treatment, in which they found that the most important network branched out from the ABCF1 gene, a gene controlled by the major histocompatibility complex, and placed in an SCZ-associated genetic region (Debnath et al., 2013). In 2005, Tsuang et al. found an 8-gene putative biomarker capable of discerning individuals with BD, SCZ, and controls with 95% accuracy using blood-based gene expression. Although most of the work has been placed toward purely genetic markers of treatment response (Cruceanu et al., 2011; Narasimhan and Lohoff, 2012), genetic variation alone might not explain response, suggesting that other factors are possibly involved.

### Biomarkers in Transcriptomics

Another methodology for current biomarker discovery in major psychiatric disorders is high-throughput microarray gene chips that include the whole human transcriptome and are capable of identifying mRNA abundances (eg, expression profiles). The examination for gene expression changes associated with SCZ is the most worn research path towards biomarker identification (Pickard, 2015). In one study, whole blood was acquired from 52 antipsychotic-naive SCZ patients and 49 healthy controls. Altogether, 792 differentially expressed genes were exposed through microarray analysis and the process of cell adhesion recognized as a significantly overrepresented gene ontology term within. A neural network method was then presumed in order to express a diagnostic set of genes (Takahashi et al., 2010). Another study of blood gene expression was reported in patients diagnosed with medicated SCZ subjects which were used as the basis for a diagnostic test and that was able to appropriately identify disease status in 89.3% and 70% of cases of SCZ and healthy controls, respectively (Maschietto et al., 2012).

In addition to the traditional mRNA arrays, a somewhat new and promising transcriptomic approach was established using microRNAs (miRNAs). miRNAs are small (approximately 22 nucleotides) noncoding RNAs that perform cell and tissue regulatory functions pertaining to development and homeostasis (Kocerha et al., 2009). However, since the discovery of miRNAs (Lee et al., 1993), a limited amount of miRNA studies have been shown in major psychiatric disorders (Dwivedi, 2014). Much consideration has been given to the role of miRNAs, predominantly in SCZ (Perkins et al., 2007; Beveridge et al., 2008). miRNA data have recently been placed into the Gene Expression Omnibus presenting altered miRNA expression in the frontal cortex of major depressive patients (NCBI accession number GSE17440). The number of miRNAs currently stands at 706 according to the Sanger miRBase release 13.0 miRNA database (http://microrna.sanger.ac.uk/sequences) (Griffiths-Jones et al., 2008). This improvement in utilization and discovery is in part a result of recent development of miRNA microarrays by Illumina, Affymetrix, Applied Biosystems, and Agilent that are accomplished of searching all known human miRNAs at one time.

### Biomarkers in Proteomics

In recent years, proteomics has appeared as a viable approach used not only to recognize novel diagnostic and therapeutic biomarkers but also to investigate clinical diagnostics and drug development for psychiatric disorders (Patel, 2014; Sethi et al., 2015). Proteomics-based technologies for biomarker discovery have been encouraging, because changes in protein expression and its abundance, structure, or function can be used as indicators of pathological abnormalities prior to expansion of clinical symptoms of neuropsychiatric disorders.

Genomics-based tools have provided important perceptions in neuroscience and psychiatry research, but when it comes to clinical use, it has been unsuccessful in progressing the diagnostic and therapeutic options in brain disorders (Hünnerkopf et al., 2007). On the contrary, using protein identification based on high-throughput mass spectrometric (MS) analysis, it is likely to unravel signal transduction pathways and complex interaction networks on the level of proteins (Hünnerkopf et al., 2007). Research using the proteomics approach have largely enhanced our understanding of psychiatric disorders and identifyied its relevant biomarkers. Currently, new technologies like proteomics-based approaches have made it possible to look into new perceptions in context with neuropsychiatric disorders, hence overwhelming standard targeted approaches (Martins-de-Souza et al., 2010a, 2010b). Quantitative and qualitative identification of protein patterns in postmortem brain tissue, cerebrospinal fluid (CSF), plasma, or serum using proteomic tools has improved the knowledge about etiology and path mechanisms of psychiatric diseases (Hünnerkopf et al., 2007). Interestingly, this approach established the first blood-based examination to aid in SCZ diagnosis, based on the identification of a set of molecular biomarker assays (Martins-de-Souza et al., 2012). Recently, proteomic analysis of first-episode SCZ patients after risperidone treatment in plasma samples investigated significant changes in apolipoprotein A-I and guanine nucleotide binding protein, alpha stimulating and proposed that apolipoprotein A-I might be a novel biomarker related to metabolic side effects in first-episode SCZ patients treated with risperidone (Song et al., 2014).

A number of preclinical studies have also suggested that the brain-derived neurotrophic factor (BDNF), a well-established neurotrophin, plays an important role in the pathophysiology of numerous psychiatric disorders (Teche et al., 2013; Munkholm et al., 2014; Akyol et al., 2015) and regulates neuronal growth, survival, and function of the adult brain (Yukimasa et al., 2006; Calabrese et al., 2014). However, clinical investigations of BDNF in psychiatric disorders is contradictory. Some reports have stated reduced blood BDNF levels in patients with SCZ (Yang et al., 2011; Akyol et al., 2015; XY Zhang et al., 2015), BD (Machado-Vieira et al., 2007; de Oliveira et al., 2009), and MDD (Sen et al., 2008; Bocchio-Chiavetto et al., 2010; Polyakova et al., 2015), whereas others have confirmed opposite findings (Durany et al., 2001; Jevtović et al., 2011; Munkholm et al., 2014). Nevertheless, some meta-analysis of case-control studies examined changes in BDNF following pharmacologic treatment in psychiatric disorders (Goldstein and Young, 2013; Pagsberg et al., 2014; Yan et al., 2014). Additional studies are needed to examine whether BDNF can inform our understanding, treatment, and prevention of aforesaid psychiatric disorders.

### Biomarkers in Metabolomics

Metabolomics, the latest -omics strategy, offers powerful tools for describing perturbations in metabolic pathways and networks in human disease. Metabolomics has the potential to map early biochemical changes in disease and hence provides an opportunity to develop predictive biomarkers that can trigger earlier interferences (Kaddurrah-Daouk et al., 2008). In fact, metabolomics seems promising for the diagnosis and identification of key metabolic features that describe certain pathological and physiological states (Mamas et al., 2011). Recent applications of metabolomics cover widespread areas, including: disease diagnosis, drug discovery and development (Gomase et al., 2008), pharmacometabolomics and personalized medicine (Kaddurah-Daouk et al., 2008), nutrigenomics (Ryan and Robards, 2006), and metabolic engineering/ biotechnology (Buchholz et al., 2002). The use of metabolomics in the examination for novel biomarkers in different clinical areas is based on the hypothesis that diseases cause disruption of biochemical pathways leading to a metabolic fingerprint characteristic of the site and nature of the disease (Lindon et al., 2003). In fact, metabolomic signatures have already been reported for several diseases (Table 1), including MDD (Huang and Lin, 2015), SCZ (Paredes et al., 2014; Pickard, 2015), cardiovascular and coronary artery disease (Rizza et al., 2014), diabetes (M Zhang et al., 2015), BD (McIntyre et al., 2014), drug addiction (Dinis-Oliveira, 2014), and also cancers (Xiang et al., 2015).

**Table 1.** Representation of Pathways/Functions of Metabolites/Possible Biomarkers Identified in Animal Model and Human-Based Studies of Various Neuropsychiatric Disorders

**Neuropsychiatric** **Disorders**		**Model/** **Subject**	**Tissue, Blood, Cells**	**Metabolites identified** **(possible biomarkers)**	**Pathways Involved/ Functions**	**References**
Bipolar disorder		Human studies	Plasma	3-Methoxy- 4 -hydroxyphenylglycol	Mood disorder, mitochondrial function and networks	Kurita et al., 2015
				Phenyllactic acid, phenylvaleric acid, LPC (16:1), deoxytetradecasphingenine, deoxytetradecasphinganine, decanamide, pentadecatetraenal, dimethyldioxododecatrienal, hexadienoic acid, hexadienoic acid	Mitochondrial function and networks	Villaseñor et al., 2014
				L-Proline, L-isoleucine, L-ornithine, L-glutamine, L-alanine, L-threonine, glycine, L-serine, D-serine, L-glutamate	Neurotransmitter and receptor function, energy metabolism	Lorenzo et al., 2013
			Postmortem brain tissues (White matter, Gray matter)	Myo-inositol, creatine, glutamate, lactate, phosphocholine	Energy metabolism, lipid metabolism, membrane phospholipids	Lan et al., 2009
			Brain tissues (Hippocampus, Striatum)	Myo-inositol, creatine, glutamate/ glutamine, glutamine, GABA, lactate, scyllo-inositol, phosphocholine, phosphoethanolamine, ethanolamine	Energy metabolism, glutamate signaling, inhibitory neurotransmission, inositol signaling, lipid metabolism	Lan et al., 2009
		Animal studies	Serum	Glycoprotein lipids, acetate, choline, myo-inositol, glutamate, glutamine	Lipid metabolism, amino acid metabolism	Sussulini et al., 2009
		Human studies	Postmortem brain tissue (Dorsolateral prefrontal cortex)	N-acetylaspartate	Lipid synthesis and myelination	Brambilla et al., 2005
Schizophrenia		Animal studies	Brain tissues	Sphinganine, N-acetylornithine, leucine, adenosine diphosphate, myelin, N-acetyl-aspartyl-glutamate	Sphingolipid metabolism, arginine metabolism synaptic plasticity, neuroprotection	McClay et al., 2015
			Plasma	Phospatidylinositol, proline-asparagine dipeptide, glycoursodeoxycholic acid, malic acid	Cell membrane integrity, lipid metabolism	Mapstone et al., 2014
		Human studies	Serum	γ-Glutamylcysteine, linoleic acid, arachidonic acid, D-serine, 3-hydroxybutyrate, glutathione, 5-hydroxytryptamine, threonine, tyrosine, D-lactate, tryptophan, kynurenine, glutamate	Oxidative stress metabolism, glyoxalase pathway	Fukushima et al., 2014
			Peripheral blood mononuclear cells (PMCs)	Pyroglutamic acid, sorbitol, tocopherol-α	Energy metabolism, oxidative stress metabolism, neurotransmitter metabolism	Liu et al., 2014
			Postmortem brain tissue	Glucose, insulin	Glucose metabolism, insulin signaling pathways	Harris et al., 2013
			Serum	Glycerate, pyruvate, glutamate, 2-hydroxybutyrate, myo-Inosital	Fatty acids metabolism, carbohydrates metabolism, amino-acid metabolism, inositol phosphate metabolism	Yang et al., 2013
			Plasma	Ornithine, arginine, glutamine, histidine, PC ae C38:6	Glutamine and arginine metabolism, nitrogen compound biosynthetic process, learning memory behavior	He et al., 2012
			Serum	Glucose, 1,3-bisphosphoglycerate, lactate, citrate, α-ketoglutarate, allantoin, uric acid, γ-tocopherol, N-acetylaspartate, aspartate, glycine, tryptophan, myo-inositol, glucuronic acid, linoleic acid, oleic acid, stearic acid, palmitic acid, glycerol, cholesterol, lactobionic acid, Erythrose	Energy metabolism, antioxidant defense systems, neurotransmitter metabolism, fatty acid biosynthesis, phospholipid metabolism	Xuan et al., 2011
			Postmortem brain tissue (Dorsolateral prefrontal cortex)	N-acetylaspartylglutamate, lipid condent of myelin	NAA metabolism, myelin synthesis	Tkachev et al., 2007
			CSF	Lactate, glucose, glutamine, citrate	Glucose metabolism	Holmes et al., 2006
Drug of Abuse	Alcohol	Animal models	Brain tissues (Cortical, striatum)	Dopamine, Met-enkephalin	Energy metabolism	Meinhardt et al., 2015
	Morphine		Plasma	3-hydroxybutyric acid, L-tryptophan, cystine, n-propylamine	MOR addiction, starvation-induced hypoglycemia	Zaitsu et al., 2014
	Cocaine			Threonine, cystine, spermidine, n-propylamine	Stress response, immune response	Zaitsu et al., 2014
	Nicotine		Brain tissues (Nucleus accumbens, striatum)	Glutamate, tryptamine, glucose, lactate, creatine, L-methylhistidine, glutamine, profine, α-ketogultaric acid	Neurotransmitter disturbance, energy metabolism imbalance, membrane and amino acids disruptions	Li et al., 2014a
	Nicotine + cocaine		Brain tissues (nucleus accumbens, striatum, hippocampus, prefrontal cortex)	Glutamate, acetylcholine, tryptamine, glucose, lactate, creatine, 3-hydroxybutyrate, nicotinamide-adenine dinucleotide, glutathione, taurine, phosphocholine	Neurotransmitter disturbance, energy metabolism dysregulation, anti-oxidation and membrane function disruptions, amino acid metabolism imbalance	Li et al., 2014b
	Methamphetamine		Brain tissues	Homocarnosine, 4-guanidinobutanoate, pantothenate, myo-inositol	Psychomotor sensitization, seizure control, transamination, anxiety-related phenotypes	Adkins et al., 2013
	Heroin		Serum	Tryptophan, 5-hydroxytryptamine	Energy metabolism	Zheng et al., 2013
	Cocaine		Brain tissues (Frontal cortex, thalamic, striatal)	Serotonin, norepinephrine, glucose, dopamine, DOPAC, 5-HIAA	Glucose metabolism, biogenic amine metabolism	Kaplan et al., 2013
			Liver, serum	N-hydroxybenzoylnorecgonine, hydroxybenzoylecgonine, α-glucoside of N-hydroxybenzoylnorecgonine, aryl hydroxy glucuronides, alanine aminotransferase	Cocaine metabolism, Oxidative metabolism	Yao et al., 2013
			Brain tissues (nucleus accumbens, striatum)	Glutamate, GABA, creatine, taurine, N-acetylaspartate, choline, phosphocholine, glycerol, leucine, L-lycine, cysteine	Neurotransmitter disturbance, mitochondrial dysregulation, oxidation stress alteration, membrane function disruptions, amino acid metabolism	Li et al., 2012
	Methamphetamine		Plasma	5-Oxoproline, saccharic acid, uracil, 3-hydroxybutyrate (3-HB), adipic acid, glucose, glucose 6-phosphate, fructose 1,6-bisphosphate, fumarate	Energy metabolism, fatty acid metabolism	Shima et al., 2011
	Cocaine	Human studies	Plasma	Anthranilate, N-methylserotonin, N-acetyl serotonin, hypoxanthine, xanthine, guanine	Tryptophan metabolism, purine metabolism	Patkar et al., 2009

Another aspect of metabolomics as a tool for discovery of biomarkers is its ability to understand the relationships and interactions between metabolic state of an individual and environmental aspects (diet, lifestyle, gut microbial activity, and genetics) under a particular set of conditions (Nicholson, 2006; Holmes et al., 2008a; Quinones and Kaddurah-Daouk, 2009) and provide metabolic phenotyping (metabotyping) in health and disease (Holmes et al., 2008b; Nicholson et al., 2012). For example, GWAS have found associations between genotype variation and disease phenotypes (Adamski and Suhre, 2013), and, analogously, the metabolome wide association (MWAS) has revealed associations of metabolic phenotypes with disease risk in the general population and relates these metabotypes to disease risk factors (Holmes et al., 2008a, 2008b). The main advantage of the MWAS approach is that the resulting biomarkers are genuine metabolic endpoints, and investigations into these pathway perturbations may yield new therapeutic targets. Therefore, MWAS studies have the potential to provide new insights into disease mechanisms and pathophysiology that may ultimately lead to new drug targets.

Overall, metabolomics-based biomarkers should prove to be useful for disease diagnosis and screening, therapeutics toxicity and efficacy assessment, patient stratification, drug discovery, and monitoring of patient response to treatment (Griffiths et al., 2010).

### Biomarkers in Lipidomics

Lipidomics is the comprehensive analysis of molecular lipid species with their quantitation and metabolic pathways (German et al., 2007). Since lipids maintain a diversity of biological functions in the processes of life such as formation of cellular membranes, energy storage, and cell signaling, they can be projected to reflect much of the metabolic status in health and disease (Gross and Han, 2006; Zhao et al., 2015). Up to now, several studies have revealed that lipidomics seems to be crucial in determining novel lipid molecular species that function as potential biomarkers in many lipid-related diseases. Comprehensive applications of lipidomics in the discovery of potential lipid biomarkers have been carried out for certain metabolic diseases such as obesity (Yetukuri et al., 2007), diabetes (Han et al., 2007), cardiovascular disease (Brindle et al., 2002), and cancers (Tung et al., 2008). One of the most extensively used lipid biomarkers has been cholesterol, which, in the form of total blood cholesterol and/or high density lipoprotein cholesterol, has been used in risk calculations for cardiovascular disease for more than 50 years (Meikle et al., 2009).

In one study, Kaddurah-Daouk and colleagues (2007) used a specialized lipidomics platform and found alterations in different lipid classes (phosphatidylcholine, phosphatidylethanolamine, triacylglycerol) were found in the plasma of SCZ patients after 2 to 3 weeks of treatment with atypical antipsychotic drugs. A recent study has also demonstrated that significant downregulation of several n3 and n6 polyunsaturated fatty acid compositions in phosphatidylethanolamine and phosphatidylcholine lipid classes in the blood plasma of first-episode SCZ patients (McEvoy et al., 2013). These changes in lipid metabolism could indicate a metabolic vulnerability in patients with SCZ that occurs early in the development of the disease. Apart from applications in human diseases, the strategy of lipidomics-driven biomarker discovery has also been used in fields of nutrition and health necessary for health promotion and disease prevention (Draisma et al., 2008).

### Biomarkers in Epigenetics

Epigenetics is the study of long-lasting modification of nuclear DNA (eg, methylation or nucleosome modification) that is often influenced by the environment and displays itself as changes in gene expression (Pickard, 2015). The new data fortune and knowledge relating to epigenetics obtained in recent years highlights an exciting future for epigenetics research. As more epigenetic marks are associated with specific diseases, tools can be advanced to improve diagnosis and assessment of severity of disease. There is also a great interest in therapeutic epigenetics. Several drugs, such as DNA methyltransferase inhibitors and histone deacetylase inhibitors, are already used in cancer treatment (Esteller, 2007).

The application of epigenetics for the detection and diagnosis of psychiatric disorders is a new and potentially promising area of research (Nishioka et al., 2012). Several lines of evidence obtained from such research suggest that the *RELN* gene, encoding reelin, is epigenetically altered in patients with psychosis, resulting in reduced expression of reelin (Peedicayil, 2007). Reelin is an extracellular matrix glycoprotein that is involved in guiding neurons and radial glial cells to their correct positions in the developing brain and in neurotransmission, memory formation, and synaptic plasticity in the adult brain (Fatemi, 2005).

### Biomarkers in the Immune System

Considered as the hormones of the immune system, cytokines play a significant role in infection and inflammation and are key signaling molecules of the immune system that exert effects in the CNS and immune system. Modifications in the cytokine network could be related to the pathophysiology of neuropsychiatric disorders or even its etiology. Numerous hypotheses exist regarding aberrant levels of proinflammatory cytokines in the serum, plasma, and CSF of patients with SCZ and major mood disorders (Dowlati et al., 2010; Miller et al., 2011; Kalia et al., 2015; Pickard, 2015). Potvin et al. (2008) suggested a T helper Type 1/T helper Type 2 (Th1/Th2) disproportion hypothesis wherein an increase in in vivo peripheral levels of intereulin-1RA (IL-1RA), soluble interleukin-2R (sIL-2R), and IL-6 and a decrease in in vitro IL-2 secretion in SCZ patients provide the evidence of establishment of an inflammatory syndrome in SCZ. Kanba and Kato (2014) pronounced the microglial hypothesis that activated CNS micorglia release proinflammatory cytokines and free radicals that cause abnormal neurogenesis, neuronal degradation, and white matter abnormalities contribute to the pathophysiology of SCZ.

There are increased concentrations of IL-6 in both patients with SCZ and first-episode patients, suggesting that immune system abnormalities may be endophenotype of SCZ. However, no difference in IL-6 levels compared with controls has been found in outpatients with stable medication and in patients with treatment-resistant psychosis (Miller et al., 2011). Recently, García-Bueno et al. (2014) used a set of biochemical and molecular analyses to identify inflammatory pathologies in plasma and peripheral blood mononuclear cell samples from 117 patients recently diagnosed with SCZ and 106 matched controls. For NFκB (increased), iNOS (increased), COX2 (increased), IκBα (decreased), and PPAR (decreased), alterations were all indicative of an active inflammatory response. In another study, IL-1, IL-6, and TNF cytokine networks were activated in SCZ and BD patients; however, only about one-half of the studies were able to find the activation (Brietzke et al., 2009; Kim et al., 2009; Song et al., 2009).

## Metabolomics Overview

Metabolomics in today’s world carries on its shoulders the obligation of providing a detailed picture of metabolic pathways and their mechanisms, whether they are in humans, animals, or plants. The word origin is from the Greek *meta* meaning change and *nomos* meaning a rule set or set of laws (Crockford et al., 2008). Metabolomics (also known as metabonomics or metabolic profiling) is the “systematic study of the unique chemical fingerprints that specific cellular processes leave behind,” precisely, the study of their small-molecule metabolite (<1500Da) profiles (Daviss, 2005). The metabolome denotes the collection of all metabolites in a biological cell, tissue, organ, or organism, which are the end products of cellular processes (Jordan et al., 2009). Whereas mRNA gene expression data and proteomic analyses do not state the entire story of what might be happening in a cell, metabolic profiling can give an instant snapshot of the physiology of that cell. Even though the metabolome can be defined readily enough, it is not currently promising to analyze the complete range of metabolites by a single analytical method. In January 2007, researchers at the University of Alberta and the University of Calgary finished the first draft of the human metabolome. They assembled approximately 2500 metabolites, 1200 drugs, and 3500 food components that can be presented in the human body, as described in the literature. This confirmation, open at the Human Metabolome Database (www.hmdb.ca) and based on analysis of information existing in the current scientific literature, is far from complete (De Luca and St Pierre, 2000). Thus, metabolomic supplements data obtained from other fields such as genomics, transcriptomics, and proteomics, adding a final piece to a systems approach for the study of disease pathophysiology, biomarker identification, and drug action (Quinones and Kaddurah-Daouk, 2009).

Recent developments in metabolomics are usually based on fast, reproducible, selective, and sensitive procedures and technologies such as gas chromatography-MS (GC-MS), capillary electrophoresis-mass spectroscopy, liquid chromatography-MS (LC-MS), and magnetic resonance spectroscopy (Zheng et al., 2013). Other techniques, such as electrochemistry or Fourier Transform Infrared Spectroscopy, have also been presumed, but their application is restricted by the lack of detailed structural information that they deliver. Generally, nuclear magnetic resonance is a nondestructive technique and in spite of the overlapping chemical shifts for some metabolites, it is generally highly effective for structural explanation (Beckonert et al., 2007; Euceda et al., 2015). Besides the analytical technique, metabolomics also uses multivariate statistical analyses (eg, principal component analysis, partial least-squares discriminate analysis, orthogonal partial least-squares discriminate analysis, clustering) to study patterns in the data (without bias) that display the maximum variance (Le Gall, 2015). Preferably, metabolomics will ultimately contribute a comprehensive map of the regulation of metabolic pathways and hence of the interaction of proteins encoded by the genome with environmental factors, including drug exposure.

## Metabolic Signatures in Psychiatric Disorders

We have started to explore global metabolic prevalence and metabolic perturbations in psychiatric diseases. We attempt to ascertain biomarkers for disease, disease progression, and response to therapy and define pathways implicated in psychiatric disorders such as BD, SCZ, and addictive disorders. Table 1 represents pathways/functions of metabolites/possible biomarkers identified in animal model and human-based studies of various neuropsychiatric disorders. The examples provided below show how the use of advanced metabolomic platforms permits a global and integrated analysis of biochemical pathways and metabolic changes occurring in a disorder. Preferably, this global mapping of biochemical abnormalities would facilitate understating disease pathogenesis and the identification of clinically relevant biomarkers.

### Metabolomics in BD

BD is a severe and debilitating psychiatric condition characterized by the alternating mood states of mania and depression. The pathophysiology of the disorder and the mechanism of action of therapies used for its treatment remain poorly understood (Scola and Andreazza, 2014). Lan and colleagues (2009) identified increased levels of glutamate, creatine and *myo*-inositol in postmortem brain tissue of BD patients as well as a decreased ratio of glutamate/glutamine and increased level of and *γ*-aminobutyric acid in rat brain tissue after chronic treatment with valproate and lithium, respectively, suggesting that the equilibrium of excitory/inhibitory neurotransmission is central to the disorder.

In another study, a plasma metabolomic analysis of BD patients who had received ketamine in a placebo-controlled crossover study showed differences in distinct biochemical between responsive and nonresponsive patients that were due to alterations in the mitochondrial β-oxidation of fatty acids, suggesting disease-related dysregulation of mitochondrial function and networks (Diazgranados et al., 2010; Zarate et al., 2012; Villaseñor et al., 2014).

Recently, a naturalistic study in 2 patients with BD type I was shown to determine whether biological markers (monoamine metabolites and BDNF) are related with the switch between depressive and manic states. These data suggested that the plasma level of 3-methoxy-4-hydroxyphenylglycol, which is related to noradrenaline levels in the brain, could be used as a biomarker of mood states in BD I (Kunita et al., 2015).

### Metabolomics in SCZ

Several metabolomics studies have recently been shown in an effort to better define pathways modified in SCZ and its treatment (Quinones and Kaddurah-Daouk, 2009; Yao et al., 2012; Yang et al., 2013). Fukushima et al. (2014) identified 13 metabolites differentially regulated in the serum of SCZ patients compared with controls and suggest that oxidative stress may be involved in the pathogenesis of SCZ.

Another application of metabolomic platforms and informatics tools has recognized changes in energy and neurotransmitter metabolism in subregions of the dorsolateral prefrontal cortex of SCZ patients (Khaitovich et al., 2008) and in animal models of antipsychotic drug treatment (McLoughlin et al., 2009). Similarly, an interesting metabolomic study on postmortem tissue offers support to the concept that aberrations at the level of glutamatergic neurotransmission and myelin synthesis play a significant role in SCZ (Tkachev et al., 2007). However, most global profiling studies using postmortem brain tissue have been performed on subjects who have been treated with varying lifetime antipsychotic medication doses (Halim et al, 2008; Chan et al., 2011).

A recent metabolomic study evaluated serum samples from those with diagnoses of primary psychotic disorder (n=45), other nonaffective psychosis (n=57), affective psychosis (n=37), and matched healthy controls. Increases in saturated triglycerides, proline, glutamate, and lactate were identified with a highly significant result for proline seemingly limited to a diagnosis of SCZ. The lipid/glutamate profile fits with an energy metabolism dysfunction in SCZ with compensatory upregulation of fatty acid/ketone body metabolism (Orešič et al., 2011).

Still, additional support for this pathology comes from a metabolomics study of 112 SCZ patients and 110 healthy subjects (Yang et al., 2013). Training and test sets detected glycerate, pyruvate, glutamate, 2-hydroxybutyrate, and myo-inostiol. A combined classifier set of glycerate, eicosenoic acid, 2-hydroxybutyrate, pyruvate, and cysteine profiles was found to be 90% accurate in diagnosing SCZ in the test set.

### Metabolomics in Addictive Disorders

Several studies are ongoing to estimate signatures in addicts who use drug of abuse. Mapping these metabolic “signatures” can offer new understandings into addictive mechanisms and potentially identify biomarkers and therapeutic targets. Initial outcomes suggested that neurotransmitter pathways, purine pathways, and pathways concerned in oxidative stress all seem to be affected by cocaine or opioids (Patkar et al., 2009). Until now, it was observed that cocaine changes the metabolism of glucose and biogenic amine differently between cerebral areas, being utmost in the thalamus for the glycolysis metabolome (Kaplan et al., 2013).

Recently, Zaitsu and colleagues (2014) examined plasma metabolic profiling in different drug-induced conditioned place preference animal models by GC-MS. They demarcated altered 3-hydroxybutyric acid, L-tryptophan, cystine, and *n*-propylamine in morphine-addicted animals. Methamphetamine addiction induced significant changes in *n*-propylamine and lauric acid, whereas threonine, cystine, and spermidine levels were significantly increased in the plasma of cocaine-addicted animals.

Further metabolomics studies in nicotine-addicted animal models comprise a ^1^H-NMR spectroscopy-based metabolomics analysis in 2 brain regions that explored the mechanism by which nicotine increased behavioral response to COC. This study showed that nicotine priming can supply a beneficial environment of metabolites for reinforcing rewarding effects of cocaine (Li et al., 2014a).

Another drug of abuse, heroin, is rapidly deacetylated in vivo (very short half-life of approximately 2–4 minutes) to an active metabolite, 6-acetylmorphine, which is subsequently slowly hydrolyzed to morphine (Dinis-Oliveira et al., 2012). Hence, 6-acetylmorphine has been used as the target metabolite to identify heroin abuse in practice, but its half-life is also short (approximately 30 minutes) to document heroin consumption. The identification of endogenous compounds that can be used as metabolic biomarkers of heroin abuse would represent an alternative approach of significant importance to detect hidden effects. Zheng et al. (2013) recognized tryptophan, 5-hydroxytryptamine, and 5-hydroxyindoleacetate as potential biomarkers of long-term heroin addiction.

## Analytical Tools for Metabolome Analysis

Metabolomics tools allow us to study the metabolome, the repertoire of small molecules present in cells and tissue (Tyagi et al., 2010). Hundreds to thousands of metabolites can be separated and measured in samples of interest such as plasma, CSF, urine, or cell extracts using a diversity of commonly used metabolomics platforms such as NMR, GC-MS, LC-MS, and liquid chromatography electrochemical array detection (Milne et al., 2013; A Zhang et al., 2015).

The choice of metabolomic analytical instrumentation and software is generally goal specific, as each type of instrument has definite strengths. Liquid chromatography followed by coulometric array detection, for example, has been used in the identification of signatures in amyotrophic lateral sclerosis (Dupuis et al., 2010) and Parkinson’s disease (Bogdanov et al., 2008). It is outstanding for mapping neurotransmitter (eg, dopamine and serotonin) and oxidative stress pathways. Gas chromatography in conjunction with mass spectroscopy is often used in the analysis of lipid subsets (Kaddurah-Daouk et al., 2007). LC-MS is often used to obtain the largest possible biochemical profile data subset where metabolite concentrations might cover a broad range of information with regard to disease pathophysiology (Adamowicz and Tokarczyk, 2015; Domingues et al., 2015). In addition to standard high-sample throughput applications, NMR spectroscopy is a quantitative nondestructive, noninvasive, nonequilibrium-perturbing technique that delivers comprehensive data on solution-state molecular structures, including the atomic positions of isotopic labels (eg, ^13^C, ^15^N, or ^2^H) in different isotopomers created during stable isotope tracer studies (Fan and Lane, 2008). For example, NMR-based high-throughput analysis has been successful in psychiatric patients, including MDD, SCZ, and BD patients (Holmes et al., 2006; Lan et al., 2009; Martins-de-Souza, 2014).

## Future Directions as a Tool for Biomarker Discovery and Clinical Implications

The study of metabolism at the global or -omics’ level, stated as metabolomics, is a new but rapidly growing field that has the potential to impact our understanding of molecular mechanisms of disease. It has the potential to permit mapping of early biochemical changes in disease and hence offers an opportunity to develop predictive biomarkers to major psychiatric disorders that can trigger earlier interventions.

Discovery of possible biomarkers for major psychiatric disorders will require a paradigm shift in a biomarker discovery approach. Presently, the field utilizes mostly a traditional reductionist approach in which focus is given to the examination of individual parts and their associations to a complex condition (Bousman et al., 2010). Although this approach has enhanced our understanding of major psychiatric disorders and helped in narrowing our search for possible biomarkers, it is excessively naive in its ability to provide robust biomarkers for complex phenomena in a diversity of contexts. Replication and blinded studies are required to confirm markers identified. Connecting central and peripheral changes in psychiatric disorders is crucial towards defining if and how biochemical changes in plasma are related to changes in the brain. Combining metabolomics with imaging and other -omics approaches might be powerful ways to achieve these goals.

The advent of a variety of biomarker discovery approaches moves us a step closer to identifying possible biomarkers that could revolutionize public health. Achieving this vision needs new biomarker discovery efforts that continue pushing forward with innovative and sound methodological strategies, minimizing limitations discussed here to avoid improper application of technology and over interpretation of data.

## Summary and Conclusions

Psychiatric disorders are a major problem for public health worldwide. Development of biomarkers that could potentially improve diagnosis and predict treatment response or even the development of a mental disorder in at-risk individuals is a high-priority research topic. One of the major challenges that exists even today for the clinical diagnosis of mental disorders is the phenotypical heterogeneity that probably reflects neurobiological heterogeneity. Also, there is a requirement of precise attention on rare disease research as a model to study personalized medicine approaches for small cohorts of subjects. -Omics strategies and development of clinical bioinformatics linking the identified molecular profiles with current clinical descriptions will focus on constructing a solid foundation for the molecular characterization of rare diseases for small patient populations. Longitudinal studies are needed to approve and expand on these initial findings.

In the future, metabolomics might be the instrumental tool needed to identify shared underpinnings between several psychiatric diagnoses, reveal biological bases of precise symptoms, and ultimately implement personalized care to patients with psychiatric disorders (Ozomaro et al., 2013).

## Statement of Interest

None.
